# Time Trends and Sex Differences in the Association between Diabetes and Chronic Neck Pain, Chronic Low Back Pain, and Migraine. Analysis of Population-Based National Surveys in Spain (2014–2020)

**DOI:** 10.3390/jcm11236953

**Published:** 2022-11-25

**Authors:** Rodrigo Jiménez-García, Ana López-de-Andrés, Javier de Miguel-Diez, José J. Zamorano-León, David Carabantes-Alarcón, Concepción Noriega, Natividad Cuadrado-Corrales, Napoleón Pérez-Farinos

**Affiliations:** 1 Department of Public Health and Maternal & Child Health, Faculty of Medicine, Universidad Complutense de Madrid, IdISSC, 28040 Madrid, Spain; 2 Respiratory Care Department, Hospital General Universitario Gregorio Marañón, Instituto de Investigación Sanitaria Gregorio Marañón (IiSGM), Universidad Complutense de Madrid, 28007 Madrid, Spain; 3 Department of Nursery and Physiotherapy, Faculty of Medicine and Health Sciences, University of Alcalá, 28801 Alcalá de Henares, Spain; 4 Public Health and Psychiatry Department, Faculty of Medicine, Universidad de Málaga, 29016 Málaga, Spain

**Keywords:** diabetes, pain, neck, low back, migraine, headache

## Abstract

(1) Background: To assess the time trend in the prevalence of chronic neck pain (CNP), chronic low back pain (CLBP), and migraine or frequent headache (MFH) among people with diabetes in Spain from 2014 to 2020, this study identified sex differences and compared the prevalence of these pain sites between people with diabetes and age–sex-matched non-diabetic subjects. (2) Methods: The study design included a cross-sectional and a case–control study. The data were obtained from the European Health Interview Surveys for Spain conducted in 2014 and 2020. The presence of diabetes, CNP, CLBP, and MFH was self-reported. Study covariates included sociodemographic characteristics, comorbidities, lifestyles, and pain-related variables. (3) Results: Among people with diabetes, the prevalence of CNP, CLBP, and MFH did not improve from 2014 to 2020. Women with diabetes had a significantly higher prevalence of all the pain sites analyzed than men with diabetes. After matching by sex and age, the prevalence of CNP (26.0% vs. 21.1%; *p* < 0.001), CLBP (31.2% vs. 25.0%; *p* < 0.001), and MFH (7.7% vs. 6.5%; *p* = 0.028) was higher for people with diabetes than for those without diabetes. Self-reported mental disease was independently associated with reporting the three pain sites analyzed in people with diabetes. (4) Conclusions: The prevalence of CNP, CLBP, and MFH has remained stable over time. Remarkable sex differences were found, with a higher prevalence among women than men with diabetes. Diabetes was associated with reporting in all the pain sites analyzed. Self-reported mental disease was associated with reporting CNP, CLBP, and MFH.

## 1. Introduction

Worldwide, diabetes, neck pain (NP), low back pain (LBP), and migraine are major public health problems affecting both sexes and middle and older age groups [1,2,3,4,5].

Previous studies have suggested that diabetes is associated with a higher risk of suffering from these three pains; however, thus far, no conclusive results have been obtained [6,7,8,9].

For NP and LBP, a recent meta-analysis of cross-sectional studies concluded that diabetes was a risk factor for both pain sites, with an adjusted odds ratio (OR) of 1.24 (95% confidence interval (CI) 1.05–1.47) for NP and 1.35 (95% CI 1.20–1.52) for LBP [6].

Population studies conducted in our country have confirmed these associations [10,11,12]. People with diabetes not only seem to have more frequent pain but also more severe levels of pain [10,11,12].

Among the possible causes that can explain the association of spinal pain (NP and/or LBP) with diabetes are that people with diabetes have an increased risk of cartilage inflammation, loss of muscle strength, spinal stenosis, and degenerative intervertebral disc (IVD) disease [6,7,8,13,14]. The arguments for a spurious association between spinal pain and diabetes include the confounding effect of obesity, depression, and sedentarism, conditions more frequent among people with diabetes than without [6,7,8]. 

Diabetes has also been found to co-occur with migraine [9]. Previous investigations on the association of diabetes with migraine have found that diabetes is either a risk, a protective factor, or that there is no association [9,15,16]. Different settings (hospitals, primary care centers, population surveys), sampling methods, pain definitions, study populations, sample sizes, and statistical methods can explain the lack of conclusive results [9,15,16].

Diabetes may be relevant in migraine pathophysiology because people with diabetes show changes in nerve conduction, vascular reactivity, and an elevated level of inflammatory markers [15,17]. However, as for spinal pains, the confounding effects of obesity, mental health, and unhealthy lifestyles must be considered when examining the relationship between diabetes and migraine [9,17,18,19]. 

A higher prevalence of NP, LBP, and migraine among women than men is a constant finding in the presence of diabetes [5,8,10,11,15,20,21,22,23,24,25,26]. 

The underlying mechanism that could explain these differences remains unknown, but several reasons could be suggested, such as the increase in the inflammatory response caused by estrogen or the rapid spine degeneration in women after menopause [20,23].

Investigating the association of diabetes with NP, LBP, and migraine is, therefore, important to improving the prevention and treatment and to implementing public health policies to reduce the health and economic burden derived from these conditions. The Spanish healthcare system offers universal coverage to residents and is free of charge. All health services are provided, including physical therapy for chronic pain.

The aims of this investigation were as follows: (i) to assess the time trend in the prevalence of chronic neck pain (CNP), chronic low back pain (CLBP), and migraine or frequent headache (MFH) among people with diabetes in Spain from 2014 to 2020; (ii) to identify sex differences in the prevalence of these pain sites between men and women with diabetes; (iii) to compare the prevalence of these pain sites between people with diabetes and age–sex-matched non-diabetic subjects; and (iv) to identify which sociodemographic and clinical variables were associated with reporting these pains in people with diabetes.

## 2. Materials and Methods

### 2.1. Study Design and Data Source

The study design included a cross-sectional and a retrospective observational case–control study. The data were obtained from the European Health Interview Surveys for Spain (EHISSs) conducted in 2014 (EHISS2014) and 2020 (EHISS2020). The European Health Interview Survey was started in 2008, promoted by the European Commission, with the objective of providing comparable reliable data on health status, health determinants, and healthcare uses from population surveys for all European Union member states [27]. 

EHISSs provide a representative sample of adults aged ≥ 15 years residing in households in Spain. Information was collected through a home-based personal interview from January to December 2014 for the EHISS2014. The EHISS2020 was initially intended to be conducted from July 2019 to July 2020 using the same methodology as the EHISS2014, but due to the COVID-19 pandemic lockdown period in Spain, from March 2020 until July 2020, interviews were conducted by telephone [28,29].

All the variables used in this investigation were collected with questions identically worded in both surveys. A detailed description of the methodology, questionnaires, and non-response data for the EHISS2014 and EHISS2020 can be found on the website of the Spanish National Statistics Institute (SNSI) [28].

### 2.2. Study Population and Matching Method

We included participants aged 18 years or over. To identify those persons interviewed with and without diabetes, we used the question: “Has your doctor told you that you are suffering from diabetes?” Those with an affirmative answer were considered “cases” for study purposes. For each “case”, we selected a person interviewed in the same EHISS with the identical sex, age, and region of residence who had answered “no” to the previous questions. This participant was defined as a matched “control”. In the database, if we found more than one possible matched control for a case, the control was selected randomly. Of the 1945 participants of the EHISS2014 with diabetes, 1871 could be matched (96.20%); the equivalent figures for the EHISS2020 were 2150 and 2049 (95.30%).

### 2.3. Study Variables

The dependent study variables were the self-reported presence of “CNP”, “CLBP”, and “MFH”. The questions and possible answers used to classify participants in the EHISS2014 and EHISS2020, based on being affected by these pains, are detailed in Supplementary Table S1. The variable “spinal pain” included those participants who answered affirmatively to one or both questions regarding back pain (CNP and/or CLBP). The variable “any pain site” included those who reported suffering in one or more of any of the three pain sites studied. According to the EHISS definitions, a pain is considered chronic if it lasts for at least six months [28].

Study covariates included sociodemographic characteristics, such as age, sex, educational level, and living with a partner. Pain-related variables collected were pain intensity in the last four weeks and consumption of pain medication in the last two weeks. 

To assess the health status of the participants, self-rated health over the last year and the presence of the following chronic conditions were analyzed: chronic obstructive pulmonary disease (COPD), heart diseases (heart failure and/or coronary disease), stroke, cancer, mental disease (anxiety and/or depression), and high blood pressure. Lifestyle variables included sedentary lifestyle, alcohol consumption, active smoking, and body mass index (<25, 25–29.9, and ≥30) (Table S1).

### 2.4. Statistical Analysis

The prevalence of CNP, CLBP, and MFH was estimated according to study variables for people with and without diabetes. As descriptive statistics, we provide means with standard deviations for quantitative variables and absolute numbers with relative frequencies expressed as percentages for qualitative variables. To compare quantitative and qualitative variables, we used the Student *t*-test and the chi-square test, respectively. When comparisons were conducted for cases vs. matched controls, paired Student t-tests, and the McNemar test were applied.

Logistic regression models were constructed to identify which study variables were independently associated with reporting CNP, CLBP, and MFH among participants with diabetes. The recommendation of Hosmer et al. was used for model construction [30]. The following steps were followed: (i) bivariate analysis of each independent study variable; (ii) selection of those variables with a significant association (*p* < 0.10) in the bivariate analysis and those considered important in previous research; (iii) checking the contribution of each variable included in the model using the Wald statistic; (iv) using the likelihood ratio to compare successive models as variables were included and excluded; (v) once the final model was obtained, checking the linearity between variables and two-way interactions. The results of the multivariable models are presented as adjusted odds ratios (ORs) with 95% confidence intervals (95% CIs).

The statistical software used was STATA 14.0 (StataCorp. 2015. Stata Statistical Software: Release 14. StataCorp LP.: College Station, TX, USA).

### 2.5. Ethical Aspects

The databases of the EHISS2014 and EHISS2020 can be freely downloaded from the website of the Spanish Ministry of Health [31]. According to Spanish law, for free and public access to anonymous data collected by the health authorities, the approval of an ethics committee is waived.

## 3. Results

The total number of individuals with self-reported diabetes analyzed was 3920 (1871 in the EHISS2014 and 2049 in the EHISS2020). The distribution of the study population by study variables according to the year of survey is shown in Table 1. In both surveys, the mean ages were significantly higher among people with diabetes than among participants without this condition (68.61 years vs. 51.71 years, *p* < 0.001, in the EHISS2014; and 70.23 years vs. 53.84 years, *p* < 0.001, in the EHISS2020), and women were slightly over-represented in the sample.

The crude prevalence of CNP (27.9% vs. 24.3; *p* = 0.010), CLBP (33% vs. 29.4; *p* = 0.013), spinal pain (39.4% vs. 34.5%; *p* = 0.001), MFH (8.6% vs. 6.9%; *p* = 0.043), and “any pain site” (41.9% vs. 36.8%; *p* = 0.001) decreased from 2014 to 2020. Additionally, the intensity of pain in the last four weeks was significantly lower in 2020 (Table 1). 

### 3.1. Sex Differences in the Prevalence of Pain between Men and Women with Diabetes

**Figure 1:**
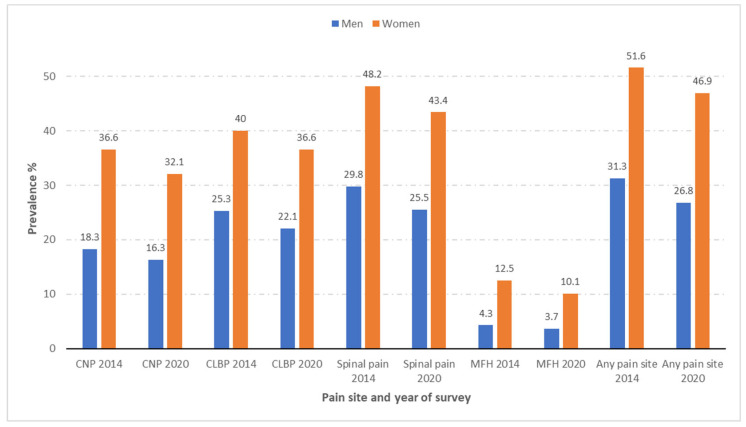
Prevalence of chronic neck pain (CNP), chronic low back pain (CLBP), spinal pain, and migraine or frequent headache (MFH), and any pain site according to sex among people with self-reported diabetes included in the European Health Interview Surveys for Spain (EHISSs) conducted in 2014 and 2020.

Shown in Figure 1 is the prevalence of CNP, CLBP, spinal pain, MFH, and “any pain site” according to sex among people with diabetes included in the EHISSs conducted in 2014 and 2020. As can be seen in the figure, women with diabetes had a significantly higher prevalence of all the pain sites analyzed than men with diabetes in both surveys (all *p* < 0.001). It is remarkable that around half of the women with diabetes reported at least one pain (51.6% in the EHISS2014 and 46.9% in the EHISS2014), with men showing a 20% lower prevalence. MHF was the pain with the lowest figures for women and men with diabetes in both surveys, with 12.5% and 10.1% for women and 4.3% and 3.7% for men in 2014 and 2020, respectively. However, this type of pain was almost three-fold higher among women. CLBP was reported by 40% of women and 25.3% of men in 2014, and by 36.6% of women and 22.1% of men in 2020. Even though this was the most frequent pain site, the proportional difference between both sexes was the lowest (58% in 2014 and 66% in 2020). The prevalence of CNP was twice as high among women with diabetes in 2014 (36.6% vs. 18.3%) and 2020 (32.1% vs. 16.3%) when compared to men with diabetes (both *p* < 0.001). 

The prevalence of all the types of pain described was significantly higher in women than in men in both the EHISS2014 and the EHISS2020.

### 3.2. Differences in the Prevalence of Pain between People with Diabetes and Age–Sex-Matched Non-Diabetic Subjects

The comparison of the prevalence of CNP, CLBP, and MFH between subjects with diabetes and sex–age-matched subjects without diabetes according to sociodemographic variables and pain characteristics can be seen in Table 2. Once the two surveys were joined, the prevalence of CNP (26.0% vs. 21.1%; *p* < 0.001), CLBP (31.2% vs. 25.0%; *p* < 0.001), and MFH (7.7% vs. 6.5%; *p* = 0.028) was significantly higher for people with diabetes than among age–sex-matched non-diabetic participants. The frequency of the three pain sites was significantly higher among people with diabetes than among controls without diabetes when the populations were stratified by sex, age, educational level, and living with partner.

The prevalence of pain sites according to clinical variables and lifestyles among subjects with diabetes and matched controls is shown in Table 3. For all the categories of the variables shown in Table 3, the prevalence of CNP, CLBP, and MFH was always significantly higher among people with diabetes than among their matched controls without diabetes.

### 3.3. Variables Associated with CNP, CLBP, and MFH in People with Diabetes

Among people with diabetes, the frequency of self-reported CNP and CLBP increased with age, whereas for MFH, the highest prevalence was observed in the youngest age group. For the three pain sites analyzed, lower educational level and use of pain medications were associated with a significantly higher prevalence (Table 2). 

As can be seen in Table 3, in the diabetes population, a remarkably high prevalence of CNP was found for those with concomitant mental disease (47.1%), COPD (42.8%), fair/poor/very poor self-rated health (34.4%), and BMI ≥ 30 (30.3%). On the other hand, the lowest frequencies were reported for those with “very good/good” self-reported health (11.7%) and those who do not have a sedentary lifestyle (19.5%). Among people with diabetes and CLBP, the highest prevalence was observed among those with a mental disease (54.6%), COPD (49.5%), and heart disease (44.8%), and the lowest was observed in those with “very good/good” self-reported health (13.7%). Finally, for MFH, mental disease (20,6%), COPD (14.5%), and cancer (13.3.8%) were the chronic conditions associated with the highest prevalence, and “very good/good” self-reported health (2.8%) was associated with the lowest prevalence.

The results of the multivariable logistic regression model for identifying variables independently associated with each of the three pain sites analyzed are shown in Table 4. After adjusting for possible confounders, women with diabetes had a significantly higher risk of reporting CNP (OR 1.78; 95% CI 1.47–2.16), CLBP (OR 1.19; 95% CI 1.01–1.48), and MFH (OR 2.12; 95% CI 56–2.82) than men with diabetes. 

Furthermore, “fair/poor/very poor” self-reported health, mental disorder, and use of pain medication were variables associated with the three pain sites.

The predictors of CNP also included COPD, concomitant CLBP, and concomitant MFH. Older age, COPD, higher BMI, and concomitant CNP or MFH increased the probability of reporting CLBP. In addition to the variables previously mentioned, concomitant CNP and CLBP were associated with MFH.

Finally, as can be seen in Table 4, the multivariable analysis showed no significant change in the prevalence of CNP, CLBP, or MFH from 2014 to 2020.

## 4. Discussion

The main results of our investigation are as follows: (1) no improvement was observed in the prevalence of pain among men and women with diabetes from the previous survey conducted six years before; (2) important sex differences in the prevalence of these pains were found, with a higher prevalence and severity among women than men with diabetes; (3) the prevalence of “any pain site” was very high among people with diabetes, and women and men with diabetes had a significantly higher prevalence of all pain sites than age-matched individuals without diabetes; (4) among women with diabetes, worse self-reported health and self-reported mental disease were associated with reporting the three pain sites analyzed after multivariable adjustment. 

### 4.1. Time Trends in the Prevalence of Pains among People with Diabetes

Population-based studies have reported that the incidence rate of spinal pain is decreasing. According to the Global Burden of Disease (GBD), in Spain, the age-standardized incidence rate of LBP showed a downward trend from 1990 to 2019, with an estimated annual percentage change (EAPCs) of −0.84% (95% CI, −0.96 to −0.72) among men and −0.15 (95% CI, −0.21 to −0.09) among women [3]. For both sexes, NP decreased by −0.3% (95% CI, −0.43 to −0.18) [32]. The equivalent figures for migraine were −0.098% for men and −0.12% for women [33].

In the USA, analysis of the National Health Interview Survey showed that the overall age-adjusted prevalence of migraine or severe headache in US adults remained remarkably stable from 2005 to 2018 [25]. In Spain, studies conducted with health surveys reported that the prevalence of NP and LBP increased from 2008/9 to 2011/12 (7.86 vs. 8.56% and 5.18 vs. 5.44%, respectively), and the prevalence of migraine also rose in the first decade of the 21st century (2003–2012) [34,35].

Regarding the trends among people with diabetes in Spain, a very recent report using the Spanish National Survey for 2017 found that the prevalence of NP and LPB was very similar to that found in our investigation, confirming the lack of improvement over time [10].

Even if effective strategies and improvements in therapies exist to reduce the incidence and alleviate spinal pain and migraine, many people who are affected, including those with diabetes, do not seem to be benefiting from this knowledge [10,25,32,33,35]. 

### 4.2. Sex Differences in the Prevalence of Pains among People with Diabetes

A remarkable result of our investigation was the higher prevalence of all pain sites among women than men with diabetes. The figures were around twice as high for spinal pain and almost three-fold for MFH. This significantly higher presence of self-reported pain among women was frequently found in previous studies conducted in Spain and other countries [8,10,11,12,16,24,36,37]. 

Different authors have suggested possible reasons to explain these sex differences in people with diabetes [8,12,24]. Glycemic control is poorer in women than in men with diabetes; therefore, low-grade systemic inflammation may be more severe in women than in their male counterparts. Additionally, depressive symptoms related to diabetes are more frequent among women than men, which could also contribute to the sex-related differences found [8,12,24].

In the general population, it has been seen that women have a greater awareness of the symptoms and signs of pain and perform more housework in non-ergonomic positions [5]. Additionally, female sex hormones, such as estrogen, have been associated with an increase in the inflammatory response, which may result in greater spine degeneration [20,23]. Finally, healthcare provider bias regarding chronic pain has been reported, as women seem to be diagnosed later, with more severe pain, and treated less effectively than men [38].

### 4.3. Differences in the Prevalence of Pains between People with Diabetes and Matched Controls

In our investigation, the prevalence of the three pain sites studied was high among people with diabetes and significantly higher than among matched controls without diabetes. Comparing the prevalence estimations with other studies is difficult because the questions used to collect pain information, the settings, the methodology, and the study populations usually differ between investigations [6,8,9,10,11,12,16,24,26,36,39,40]. However, spinal pain has been reported to be more prevalent among people with diabetes than the general population in previous investigations [6,8,10,11,12,24,39,40,41]. Pozzobon et al. conducted a meta-analysis of 11 observational studies, including cohort, case–control, cross-sectional, and twin control studies, and confirmed the association of diabetes with NP and LBP [6]. 

In our population, CNP was reported by 27.9% and 24.3% of people with diabetes in 2014 and 2020, and after matching with subjects without diabetes by year of survey, age, and sex, CNP was 4.9% higher (*p* < 0.001).

Among a total of 21,889 participants with diabetes included in the UK Biobank cohort, 21.1% of the participants with diabetes had neck and shoulder pain [41]. Analysis of this database found that type 2 diabetes was associated with neck/shoulder pain (adjusted OR 1.14, 95% CI 1.10–1.18) [41].

Molsted et al. compared the self-reported presence of neck and shoulder pain among 951 patients with T2DM and 2923 matched subjects without diabetes using the question: “Have you been bothered by pain in the shoulder and neck over the last 14 days?” The three possible answers were “Yes, very bothered”, “Yes, bothered a little”, or “No”. The percentages of people with T2DM who reported being “very bothered” or “a little bothered” were 52% and 31% (*p* < 0.001) [24].

In our country, cross-sectional analysis of 2096 twins showed that T2DM was associated with CNP (unadjusted OR 1.35; 95% CI 1.02 to 1.79; adjusted OR 1.37; 95% CI 1.01 to 1.85) [12].

As found for CNP, the prevalence of CLBP after matching was significantly higher among those with diabetes than among matched controls (31.2% vs. 25.0%; *p* < 0.001). Data from the United States, using the National Health and Nutrition Examination Survey for the period between 2010–2011, reported that the prevalence of CLBP was 19.8% among people with diabetes aged 20–69 years and 12.9% in adults without diabetes (*p* < 0.001) [39]. In Portugal, a national cross-sectional study using self-reported data, conducted from 2011 to 2013, also found a significantly higher prevalence of CLBP of 18.6% among those with diabetes, compared to 6.8% among those without the condition [36]. The lower rates compared to our results can be explained by the younger age groups analyzed in the USA study and the shorter time periods for pain recall considered in both investigations (3 months vs. 12 months) [36,39]. In the UK Biobank cohort, the prevalence of LBP pain was 32.6%, slightly higher than in our results [41]. In Denmark, the prevalence of LBP in the last 14 days among people with diabetes was 60%, with only 30% of age-, sex-, and region-matched subjects without diabetes reporting this pain [24]. 

Several causes may explain the association between diabetes and spinal pain [7,8,39,40,41,42,43,44,45]. Hyperglycemia and altered fat metabolism that are commonly present in diabetes can negatively affect intervertebral discs (IVDs), causing alterations in the structural composition, which may result in an increased risk of disc prolapse and back pain [42,43]. Animal experiments have found that diabetic rats have an elevated advanced glycation end product concentration and, consequently, lower values of glycosaminoglycan and water contents in discs [42]. This reduces the shock-absorbing properties of the IVDs, making them more susceptible to mechanical damage [42]. Microangiopathy caused by diabetes also causes a lower flow of oxygen and nutrients to the IVDs [44]. All these changes alter the biomechanics of the spine and contribute to spinal pain [7,42,43,44]. 

Some studies have suggested that dipeptidyl peptidase-4 (DPP-4) inhibitors may increase pain in the joints [45]. 

Unlike the findings found for spinal pain, the association between migraine and diabetes is controversial [9,15,16,37]. In our study population, people with diabetes reported MFH in a small but significantly higher proportion than matched non-diabetic subjects (7.7% vs. 6.5%; *p* = 0.028). In a previous study conducted in Spain, after multivariable adjustment, diabetes was not associated with a higher risk of migraine (adj. OR 1.06; 95% CI 0.89–1.25) [16]. 

Previous studies have reported the prevalence of migraine to be similar or higher among people with diabetes, and others found an inverse relationship between diabetes and migraine [9,15,16,37].

The relationship of diabetes with migraine seems to be inversely related to age, with a higher prevalence being found among younger subjects, decreasing with age. This trend was also seen in our population (Table 2) [15,16].

Despite the contradictory results that exist concerning the relationship between diabetes and migraine, diabetes may be relevant in migraine pathophysiology, considering that diabetic patients display changes in vascular reactivity and nerve conduction [15]. Possible mechanisms involved have been suggested [15]. Several pro-inflammatory markers, such as C-reactive protein, IL-1b, IL-6, IL-8, and TNF-α, are elevated in migraine and diabetes. Furthermore, the sympathetic nervous system and autonomic dysfunction found in people with diabetes may also explain the relationship between migraine and DM [15]. However, further studies should clarify this issue.

### 4.4. Variables Associated with Pains among People with Diabetes

Besides being a woman, four study variables were associated with a higher prevalence of NP, CLBP, and MFH. These were bad self-reported health, self-reported mental disease, use of pain medication, and the presence of concomitant pain in any other site. 

The relationship of worse self-rated health with any of the pain localizations analyzed was expected, as all these pains have important effects on the quality of life of people with diabetes and have been described before by other authors [10,11,15,16,24,46]. 

The association between mental diseases and spinal pain or migraine has been previously reported in the general population and among people with diabetes [8,10,16,26,34,35,47,48]. It is well known that diabetes has deleterious effects on mental health and suffering from depression and anxiety is a known risk factor associated with the transition from acute to chronic back pain [47,48].

The frequent use of pain medication has been reported among DM sufferers [10,11,16,46]. In our study population, almost half of the persons interviewed had consumed pain medication in the last two weeks. In the US, 78% of people with diabetes and chronic pain reported that they used pain medication on either a regular or an occasional basis [46]. 

We agree with other authors in that, among people with diabetes, the highest ORs were found for reporting pain in one site and the presence of pain in any other site [10,11,16,40,41,49]. It has been found that a higher number of pain sites results in multiple stimuli to the brain; therefore, the brain chemistry is altered, meaning that it is more likely that the pain becomes chronic [2].

Among people with diabetes, the multivariable model for LBP showed a significant association with a higher BMI, which has been previously reported in studies conducted in populations with diabetes [24,39,46,50,51,52]. Molsted et al. found that, in people with diabetes of both sexes, BMI was associated with a higher prevalence of LBP [24]. Data from the Nord-Trøndelag health study confirmed that BMI was strongly associated with both diabetes and low back pain [51,52].

### 4.5. Recommendations and Future Investigations

Even if CLBP, CNP, and MH have different etiologies, risk factors, and treatments, there are many actions that could be implemented to reduce the incidence, disability, and loss of quality of life caused by these pains [8,10,11,12,16,24,26,37,39,40,43,46,50,51,52]. Among possible interventions are screening for pain in patients with diabetes in clinical settings, creating specialized pain units, providing health education for patients and families regarding nonpharmacological treatments, and assessing occupational risk factors. Given the importance of mental health on pain in people with diabetes, the presence of depression and anxiety should be assessed on a regular basis [12]. Regarding lifestyles, exercise therapy and weight reduction should be recommended to patients with diabetes to lower their risk of spinal pain [12,15]. Among people with diabetes, physical exercise can reduce muscle mass loss, negative structural changes in the IVDs, and fat infiltration in the paraspinal muscles [53]. In people with diabetes, improvement in lifestyle can reduce the frequency of migraine attacks, probably by affecting peripheral and central mechanisms [15]. Future research should include longitudinal studies to obtain causal evidence of the associations found in cross-sectional studies and to find explanations for the sex-related differences in the association of diabetes with spinal pain and MFH. 

### 4.6. Strengths and Limitations

The main strength of this study is the use of two large national health surveys that provide a representative sample of the Spanish population, providing data on sociodemographic characteristics and lifestyle variables that are not usually recorded in clinical records. We analyzed the data of almost four thousand individuals with self-reported diabetes who were randomly selected from the Spanish general population, and not from health services, therefore, increasing the external validity of our results. Furthermore, we used a matched design to compare people with and without diabetes, avoiding the confounding effect of age and sex.

However, there are relevant limitations that should be considered. First, with the cross-sectional design that was used, the causality issue cannot be properly addressed. Second, in the multivariable analyses, many potential confounding factors were included, but the possibility of residual confounding factors cannot be ruled out. Third, even if the questions used for self-reported CNP, CLBP, and MFH were formulated using worldwide definitions of these conditions, these questions have not been validated in the EHISS.

Fourth, the validity of self-reported diabetes has not been evaluated in the EHISS. However, in a previous study conducted in our country, self-reported diabetes showed a sensitivity of over 70% and a specificity of over 95% when compared with medical records [54]. Self-reporting has been used as a valid method to determine diabetes status in Spain and different countries [6,9,10,11,16,25,39,55,56,57,58,59,60]. 

Fifth, important information on diabetes is not available in the EHISS, including the diabetes type, duration, pharmacological treatments, disabilities, and chronic complications. Given the epidemiology of diabetes in Spain, it is expected that over 96% of individuals with self-reported diabetes have type 2 [61]. This information was also lacking for the pain sites studied. Previous studies have suggested that people with diabetes taking metformin may be less likely to report back, neck, and multisite musculoskeletal pain than those not taking this medication [41]. Sixth, the validity of information obtained during interviews may be affected by recall errors or socially desirable responses, i.e., the tendency of respondents to reply in a manner that will be viewed favorably by others. Finally, the initial response rates for the EHISS 2014 and EHISS2020 were 61% and 59%, respectively, and thus the existence of a non-response bias must be considered [28]. Regarding this point, the EHISS2020 data collection method was affected by the COVID-19 pandemic, and thus the possible effect of the pandemic itself and the change in collection method on our results is unpredictable [29].

## 5. Conclusions

The prevalence of CNP, CLBP, and MFH among men and women with diabetes has remained stable over time. Remarkable sex differences were found, with a higher prevalence and severity among women than men with diabetes. The prevalence of all pains studied was very high among people with diabetes, and women and men with diabetes had a significantly higher prevalence of all pain sites analyzed than age-matched individuals without diabetes. Among women with diabetes, worse self-reported health and self-reported mental disease were associated with reporting the three pain sites analyzed after multivariable adjustment.

## Figures and Tables

**Table 1:** Distribution according to study variables of people with self-reported diabetes included in the European Health Interview Surveys for Spain (EHISSs) conducted in 2014 and 2020.

Variable	Categories	EHISS 2014		EHISS 2020		
		*n*	%	*n*	%	*p*
Sex	Women	982	52.4	1033	50.3	0.192
Age groups (Years)	18–59	448	23.9	416	20.3	<0.001
	60–69	493	26.3	495	24.2	
	70 or over	930	49.7	1138	55.5	
Educational level	No studies/primary	1507	80.4	1549	75.5	<0.001
	Secondary	167	8.9	239	11.6	
	High education	200	10.7	265	12.9	
Living with a partner	Yes	990	52.9	1065	52.1	0.589
Chronic neck pain	Yes	522	27.9	498	24.3	0.010
Chronic low back pain	Yes	619	33	603	29.4	0.013
Spinal pain	Yes	739	39.4	708	34.5	0.001
Migraine or frequent headache	Yes	161	8.6	141	6.9	0.043
Any pain site	Yes	1874	41.9	2053	36.8	0.001
Pain intensity	No pain	656	35	783	38.2	<0.001
	Light	460	24.6	502	24.5	
	Moderate	405	21.6	478	23.3	
	Severe/extreme	351	18.8	286	14	
Use of pain medication	Yes	867	47.6	969	48.7	0.484
Self-rated health	Very good/good	634	33.8	822	40	<0.001
	Fair/poor/very poor	1240	66.2	1231	60	
COPD	Yes	180	9.6	145	7.1	0.004
Heart diseases	Yes	304	16.2	337	16.4	0.870
Stroke	Yes	48	2.6	52	2.5	0.955
Cancer	Yes	64	3.4	64	3.1	0.600
Mental disease	Yes	381	20.3	331	16.1	<0.001
High blood pressure	Yes	1061	56.6	1221	59.5	0.070
Sedentary lifestyle	Yes	488	37.6	764	48.5	<0.001
Alcohol consumption	Yes	503	26.9	622	30.3	0.016
Active smoking	Yes	284	15.2	304	14.8	0.750
Body mass index	<25	499	26.7	597	29.1	0.013
	25–29.9	724	38.7	833	40.7	
	≥30	646	34.6	619	30.2	

COPD: Chronic obstructive pulmonary disease. *p* value for differences between EHISS 2014 and EHISS 2020.

**Table 2:** Prevalence of chronic neck pain, chronic low back pain, and migraine or frequent headache among subjects with diabetes and sex–age-matched subjects without diabetes according to sociodemographic variables and pain characteristics.

		Chronic Neck Pain				Chronic Low Back Pain				Migraine or Frequent Headache			
		No Diabetes *n*%		Diabetes *n*%		No diabetes *n*%		Diabetes *n*%		No Diabetes *n*%		Diabetes *n*%	
Sex ^d–i^	Men ^a–c^	237	12.4	329	17.2	326	17.1	451	23.6	73	3.8	76	4.0
	Women ^a–c^	590	29.3	691	34.3	653	32.4	771	38.3	180	8.9	227	11.3
Age groups ^d–i^	18–59 years	115	13.3	174	20.1	149	17.2	211	24.4	69	8.0	92	10.6
	60–69 years ^a–c^	198	20.0	249	25.2	216	21.9	312	31.6	62	6.3	68	6.9
	70 years or over ^a–c^	514	24.9	597	28.9	614	29.7	699	33.8	122	5.9	142	6.9
Educational level ^d–i^	No studies/primary ^a–c^	677	25.1	873	28.6	792	29.4	1037	33.9	190	7.0	255	8.3
	Secondary ^a–c^	85	15.8	79	19.5	94	17.5	87	21.4	35	6.5	30	7.4
	High education ^a–c^	65	9.4	68	14.6	93	13.5	98	21.1	28	4.1	18	3.9
Living with a partner ^d–i^	No ^a–c^	449	24.4	498	26.8	504	27.4	592	31.8	109	5.9	159	8.5
	Yes ^a–c^	375	18.1	521	25.4	473	22.8	628	30.6	144	6.9	143	7.0
Pain intensity ^d–i^	No pain ^a–c^	120	6.6	116	8.1	142	7.9	135	9.4	32	1.8	34	2.4
	Light ^a–c^	175	18.5	203	21.1	213	22.6	252	26.2	56	5.9	46	4.8
	Moderate ^a–c^	291	39.1	341	38.6	344	46.2	409	46.3	93	12.5	94	10.6
	Severe/extreme ^a–c^	240	56.1	359	56.4	277	64.7	425	66.7	71	16.6	129	20.3
Use of pain medication ^d–i^	No ^b,c^	198	11.3	246	12.4	240	13.7	298	15.1	53	3.0	57	2.9
	Yes ^b,c^	587	41.4	756	41.2	684	48.2	903	49.2	183	12.9	243	13.2
Concomitant chronic neck pain ^e–i^	No ^a,c^	-	-	-	-	361	11.6	427	14.7	119	3.8	140	4.8
	Yes ^a–c^	-	-	-	-	618	74.7	795	77.9	134	16.2	163	16.0
Concomitant chronic low back pain ^d–i^	No ^a,c^	209	7.1	225	8.3	-	-	-	-	125	4.2	127	4.7
	Yes ^a,c^	618	63.1	795	65.1	-	-	-	-	128	13.1	176	14.4
Migraine or frequent headache ^d–h^	No ^a,b^	693	18.9	857	23.6	851	23.2	1046	28.9	-	-	-	-
	Yes ^a,b^	134	53.0	163	53.8	128	50.6	176	58.1	-	-	-	-

^a^ Significant difference between diabetes sufferers and non-diabetes controls with chronic neck pain. ^b^ Significant differences between diabetes sufferers and non-diabetes controls with chronic low back pain. ^c^ Significant difference between diabetes sufferers and non-diabetes controls with migraine or frequent headache. ^d^ Significant association between the variable and chronic neck pain among people without diabetes. ^e^ Significant association between the variable and chronic low back pain among people without diabetes. ^f^ Significant association between the variable and migraine or frequent headache among people without diabetes. ^g^ Significant association between the variable and chronic neck pain among people with diabetes. ^h^ Significant association between the variable and chronic low back pain among people with diabetes. ^i^ Significant association between the variable and migraine or frequent headache among people with diabetes.

**Table 3:** Prevalence of chronic neck pain, chronic low back pain, and migraine or frequent headache among subjects with diabetes and sex–age-matched subjects without diabetes according to clinical variables and lifestyles.

		Chronic Neck Pain				Chronic Low Back Pain				Migraine or Frequent Headache			
		No Diabetes, *n*%		Diabetes, *n*%		No Diabetes, *n*%		Diabetes, *n*%		No Diabetes, *n*%		Diabetes, *n*%	
Self-rated health ^d–i^	Fair/poor/very poor	616	35.9	850	34.4	717	41.8	1023	41.4	178	10.4	262	10.6
	Very good/good	211	9.5	170	11.7	262	11.8	199	13.7	75	3.4	41	2.8
COPD ^d–i^	No ^a–c^	744	20.0	881	24.5	889	23.9	1061	29.5	231	6.2	256	7.1
	Yes ^a–c^	83	40.7	139	42.8	90	44.1	161	49.5	22	10.8	47	14.5
Heart diseases ^d–i^	No^a–c^	686	19.7	783	23.8	826	23.7	935	28.5	222	6.4	225	6.8
	Yes ^a–c^	141	31.8	237	37.0	153	34.5	287	44.8	31	7.0	78	12.2
Stroke ^d,e^	No^a–c^	806	20.8	986	25.8	955	24.7	1183	30.9	247	6.4	291	7.6
	Yes ^a–c^	21	35.0	34	34.0	24	40.0	39	39.0	6	10.0	12	12.0
Cancer ^i^	No ^a–c^	796	20.9	980	25.8	951	24.9	1173	30.9	247	6.5	286	7.5
	Yes ^a–c^	31	27.4	40	31.3	28	24.8	49	38.3	6	5.3	17	13.3
Mental disease ^d–i^	No ^a–c^	606	17.6	685	21.3	728	21.2	833	25.9	160	4.7	156	4.9
	Yes ^a–c^	221	44.8	335	47.1	251	50.9	389	54.6	93	18.9	147	20.6
High blood pressure ^d–i^	No ^a–c^	428	17.7	344	20.9	497	20.6	412	25.0	136	5.6	105	6.4
	Yes ^a–c^	399	26.4	676	29.6	482	31.9	810	35.5	117	7.7	198	8.7
Sedentary ^d–i^	No ^a–c^	284	15.9	315	19.5	341	19.1	405	25.0	92	5.1	80	4.9
	Yes ^a–c^	253	19.2	318	25.4	316	24.0	379	30.3	83	6.3	87	6.9
Alcohol consumption ^d–i^	No ^a–c^	610	24.6	794	28.4	699	28.2	941	33.6	187	7.5	264	9.4
	Yes ^a–c^	215	14.9	225	20.0	276	19.2	280	24.9	64	4.4	39	3.5
Active smoking ^d–h^	No ^a–c^	726	21.9	887	26.6	857	25.8	1064	31.9	211	6.4	264	7.9
	Yes ^a–c^	100	16.8	133	22.6	120	20.1	158	26.9	40	6.7	39	6.6
Body mass index ^d–i^	<25 ^a–c^	273	18	261	23.8	312	20.5	310	28.3	101	6.6	84	7.7
	25–29.9 ^a–c^	349	21.7	373	24.0	420	26.1	435	27.9	87	5.4	99	6.4
	≥30 ^a–c^	203	25.8	383	30.3	244	31	473	37.4	63	8.0	118	9.3

^a^ Significant difference between diabetes sufferers and non-diabetes controls with chronic neck pain. ^b^ Significant differences between diabetes sufferers and non-diabetes controls with chronic low back pain. ^c^ Significant difference between diabetes sufferers and non-diabetes controls with migraine or frequent headache. ^d^ Significant association between the variable and chronic neck pain among people without diabetes. ^e^ Significant association between the variable and chronic low back pain among people without diabetes. ^f^ Significant association between the variable and migraine or frequent headache among people without diabetes. ^g^ Significant association between the variable and chronic neck pain among people with diabetes. ^h^ Significant association between the variable and chronic low back pain among people with diabetes. ^i^ Significant association between the variable and migraine or frequent headache among people with diabetes. COPD, chronic obstructive pulmonary disease.

**Table 4:** Factors associated with suffering from chronic neck pain, chronic low back pain, and migraine or frequent headache among people with diabetes. Results of multivariable logistic regression analysis.

		Chronic Neck Pain	Chronic Low Back Pain	Migraine or Frequent Headache
		OR (95% CI)	OR (95% CI)	
Sex	Man	1	1	1
	Women	1.78 (1.47–2.16)	1.19 (1.01–1.48)	2.12 (1.56–2.82)
Age groups	15–59 years	NIFM	1	NIFM
	60–69 years	NIFM	1.44 (1.10–1.87)	NIFM
	70 years or over	NIFM	1.29 (1.03–1.72)6	NIFM
Self-rated health	Very good/good	1	1	1
	Fair/poor/very poor	1.35 (1.05–1.72)	2.07 (1.65–2.59)	1.81 (1.22–2.67)
COPD		1	1	NIFM
		1.47 (1.08–1.99)	1.26 (1.02–1.69)	NIFM
Mental disorder	No	1	1	1
	Yes	1.36 (1.08–1.72)	1.62 (1.29–2.03)	2.66 (2.04–3.47)
Body mass index	<25	NIFM	1	NIFM
	25–29.9	NIFM	1.02 (0.81–1.27)	NIFM
	≥30	NIFM	1.34 (1.07–1.68)	NIFM
Use of pain medication	No	1	1	1
	Yes	2.67 (1.84–3.98)	3.03 (2.23–4.11)	2.45 (1.62–3.89)
Concomitant chronic neck pain	No	NA	1	1
	Yes	NA	15.15 (12.54–18.29)	1.82 (1.33–2.49)
Concomitant chronic low back pain	No	1	NA	1
	Yes	15.28 (12.66–18.45)	NA	1.44 (1.05–1.98)
Migraine or frequent headache	No	1	NIFM	1
	Yes	1.78 (1.31–2.44)	NIFM	1.41 (1.02–1.93)
Year	2020	0.96 (0.81–1.12)	0.91 (0.76–1.01)	0.90 (0.77–1.02)

COPD, chronic obstructive pulmonary disease. NA, not adequate. NIFM, not included in final the model. OR, odds ratios estimated using multivariable unconditional logistic regression. CI, confidence interval.

## Data Availability

According to the contract signed with the Spanish Ministry of Health and Social Services, which provided access to the databases from the Spanish National Health Survey and European Health Survey for Spain, we cannot share the databases with any other investigator, and we have to destroy the databases once the investigation has concluded. Consequently, we cannot upload the databases to any public repository. However, any investigator can apply for access to the databases by filling out the questionnaire available at http://www.msssi.gob.es/estadEstudios/estadisticas/estadisticas/estMinisterio/SolicitudSNHSdocs/Formulario_Peticion_Datos_SNHS.pdf. (accessed on 14 September 2022). All other relevant data are included in the paper.

## Supplementary Materials

The following supporting information can be downloaded at: https://www.mdpi.com/article/10.3390/jcm11236953/s1, Table S1: Definition of variables according to the questions included in the European Health Interview Surveys in Spain conducted in years 2014 and 2020.
